# DNA barcoding as a complementary tool for conservation and valorisation of forest resources

**DOI:** 10.3897/zookeys.365.5670

**Published:** 2013-12-30

**Authors:** Angeliki Laiou, Luca Aconiti Mandolini, Roberta Piredda, Rosanna Bellarosa, Marco Cosimo Simeone

**Affiliations:** 1 Department of Agriculture, Forests, Nature and Energy (DAFNE) - Università degli Studi della Tuscia, via S. Camillo de’ Lellis, 01100 Viterbo, Italy

**Keywords:** DNA barcoding, Forest Biodiversity, Medicinal and Aromatic plants, Conservation

## Abstract

Since the pre-historic era, humans have been using forests as a food, drugs and handcraft reservoir. Today, the use of botanical raw material to produce pharmaceuticals, herbal remedies, teas, spirits, cosmetics, sweets, dietary supplements, special industrial compounds and crude materials constitute an important global resource in terms of healthcare and economy. In recent years, DNA barcoding has been suggested as a useful molecular technique to complement traditional taxonomic expertise for fast species identification and biodiversity inventories. In this study, *in situ* application of DNA barcodes was tested on a selected group of forest tree species with the aim of contributing to the identification, conservation and trade control of these valuable plant resources.

The “core barcode” for land plants (*rbc*L, *mat*K, and trnH-psbA) was tested on 68 tree specimens (24 taxa). Universality of the method, ease of data retrieval and correct species assignment using sequence character states, presence of DNA barcoding gaps and GenBank discrimination assessment were evaluated. The markers showed different prospects of reliable applicability. *Rbc*L and trnH-psbA displayed 100% amplification and sequencing success, while *mat*K did not amplify in some plant groups. The majority of species had a single haplotype. The trnH-psbA region showed the highest genetic variability, but in most cases the high intraspecific sequence divergence revealed the absence of a clear DNA barcoding gap. We also faced an important limitation because the taxonomic coverage of the public reference database is incomplete. Overall, species identification success was 66.7%.

This work illustrates current limitations in the applicability of DNA barcoding to taxonomic forest surveys. These difficulties urge for an improvement of technical protocols and an increase of the number of sequences and taxa in public databases.

## Introduction

Forests figure prominently among the world’s most important ecosystems. The importance of trees in sustaining biodiversity and habitat stability, as well as to provide a large variety of environmental services is well acknowledged. Nevertheless, the increasing human impact, the recent environmental decay, and the on-going climate change are among the main factors affecting forest communities, especially at local and regional scales within the Mediterranean basin (FOREST EUROPE, UNECE and FAO 2011). In the meantime, international market pressures call for higher quality standards. One way to convince decision-makers of the importance of conserving wild plants and habitats is to demonstrate their economic potential (Kathe 2006). The socio-economic contribution of forests to livelihood and the impact of their use on the environment are essential components of modern concepts for sustainable forest management (Arnold and Perez 2001).

Temperate and boreal forests are a traditional source, not only for timber, but also for many products that have been extracted from forests for millennia, including resin, tannin, fodder, litter, medical plants, fruits, nuts, roots, mushrooms, seeds, honey, ornamentals and exudates. Today there is an institutional rediscovery of the value of forest products and services other than timber, and the total value of Non-Wood Goods (NWGs) reported in Europe has almost tripled since 2007 (FOREST EUROPE, UNECE and FAO 2011).

Besides wood trade, Mediterranean woody flora includes numerous valuable species used as ornamentals or for secondary products processing and marketing (edibles, industrial and medicinal compounds). The option of stimulating the production of non-timber forest products has long been considered promising (Arnold and Perez 2001, Wunder 2001), and it is well illustrated in the case of Medicinal and Aromatic Plants (MAPs). In many Euro-Mediterranean countries MAPs resources are still unknown or overlooked (Lange 2006). In other countries, the necessary plant materials (roots, bark, leaves, fruits and seeds) are generally collected and sold by local people to traders and to the industry. Final products are then purchased by international exporters (WHO 2003). Forest overexploitation, product forgery and misidentifications are common risks, with the latter two usually occurring as a result of morphologically indistinguishable materials, species with similar common names, or intentional substitution of economically valuable materials by inexpensive specimens. At the same time, plant misidentification and forgery are serious threats to human health (Vanherweghem et al. 1993, Barthelson et al. 2006, Sundus 2008). The identification of herbal medicinal materials using traditional, organoleptic and chemical methods can be difficult, particularly for processed materials of a plant (Govindaraghavan et al. 2012). Also plant germplasm (seeds and seedlings) purchased for the establishment of MAPs orchards, afforestation programs, and ornamentals, may be difficult to recognize. Therefore, an accurate, universal, stable and specific method allowing non-specialists to identify the source species from a tiny amount of tissue is needed.

Molecular technology is considered a reliable alternative tool for the identification of plant species (e.g. Savolainen et al. 2000) and DNA barcoding is the latest move towards the generation of universal standards (Kane and Cronk 2008). A DNA barcode is a universally accepted short DNA sequence allowing the prompt and unambiguous identification of species (Savolainen et al. 2005), promoted for a variety of biological applications (Hollingsworth et al. 2011), including biodiversity inventories (Costion et al. 2011, de Vere et al. 2012), the identification of medicinal plants (Heubl et al. 2010), of natural health products (Wallace et al. 2012), and of tree species listed in the Convention on International Trade of Endangered Species (Muellner et al. 2011).

Based on the relative ease of amplification, sequencing, multi-alignment and the amount of variation displayed (sufficient to discriminate among sister species without affecting their correct assignation through intraspecific variation), three plastid loci are currently used in plants: *rbc*L (a universal but slowly evolving coding region), *mat*K (a relatively fast evolving coding region) and trnH-psbA (a rapidly evolving intergenic spacer) (CBOL Plant Working Group 2009). More recently, the nuclear ribosomal internal transcribed spacer (ITS) has also been suggested as an efficient barcoding locus for complex plant groups (Hollingsworth et al. 2011).

Tree taxa have peculiar biological, evolutionary and taxonomic features that are likely to constitute a challenge to species recognition through DNA barcodes, viz. the generally low mutation rate of the plastid DNA, their ability to hybridize, and their narrowly defined species limits (Petit and Hampe 2006). Nevertheless, DNA barcoding has proven its utility in several detailed studies of tree genera (Newmaster et al. 2008, Newmaster and Ragupathy 2009, Kress et al. 2009, 2010, Ren et al. 2010, Roy et al. 2010, Liu et al. 2011). In this study, *in situ* application of DNA barcoding was applied to a number of indigenous and introduced tree species in the Mediterranean area, with medicinal, ornamental, edible, industrial and conservation relevance. Taxa were analysed with the core barcode for land plants (*rbc*L, *mat*K, and trnH-psbA); ease and success to achieve correct species identification were evaluated based on the relative efficiency of each marker, data quality and representation in the GenBank/EMBL database. Our final objective is to provide a contribution to the future assemblage of a regional data/species inventory in the Mediterranean area for adequate identification, conservation and trade control of these valuable resources.

## Materials and methods

### Plant material and molecular analyses

Sixty eight trees belonging to 24 species (ten genera, nine families) were sampled in the wild (Italy, Greece and adjacent areas) and/or Botanic Gardens (Table 1). Plants were identified directly in the field. Herbarium specimens and lyophilized green tissues of the collected material were vouchered and preserved at the Mediterranean Forest DNA bank of the University of Tuscia (www.Medna-bank.eu).

**Table 1.** Sample list.

Familia	Species	Relevance	No. of samples
Pinaceae	*Cedrus atlantica*	Ornamental/afforestation	3
	*Cedrus deodara*	Ornamental/afforestation	3
	*Cedrus libani*	Ornamental/afforestation/conservation	3
Rosaceae	*Crataegus monogyna*	Medicinal/ornamental	3
	*Crataegus oxyacantha*	Medicinal/ornamental	2
	*Crataegus azarolus*	Food industry/conservation	4
	*Sorbus aria*	/	3
	*Sorbus aucuparia*	Ornamental/conservation	2
	*Sorbus domestica*	Medicinal/food industry	3
	*Sorbus torminalis*	Valuable wood industry	3
Sapindaceae	*Aesculus hippocastanus*	Medicinal/ornamental	3
	*Aesculus indica*	/	3
Oleaceae	*Fraxinus ornus*	Medicinal/food industry	5
	*Fraxinus angustifolia*	/	3
	*Fraxinus excelsior*	/	2
Adoxaceae	*Sambucus nigra*	Medicinal	5
	*Sambucus ebulus*	/	2
	*Sambucus racemosa*	/	1
Passifloraceae	*Passiflora incarnata*	Medicinal/ornamental	2
	*Passiflora edulis*	Food industry	1
Lythraceae	*Punica granatum*	Medicinal/food industry/ornamental	4
Rhamnaceae	*Ziziphus jujuba*	Medicinal/food industry	3
Aquifoliaceae	*Ilex aquifolium*	Medicinal/ornamental/conservation	4
	*Ilex latifolia*	/	1

DNA extractions were performed with the DNeasy Plant Minikit (QIAGEN), following the manufacturer’s instructions. The universal applicability of the technical analyses was considered a prerequisite for exploring the DNA barcoding potential in a practical floristic case study: uniform PCR procedures were thus performed for all taxa and barcoding loci. Genomic DNAs (ca. 40 ng) were amplified with RTG PCR beads (GE Healthcare) in 25 μl final volume according to the manufacturer’s protocol. Thermocycling conditions were as follows: 94 °C for 3 min, followed by 35 cycles of 94 °C for 30 s, 53 °C for 40 s and 72 °C for 40 s, with a final extension step of 10 min at 72 °C. Primers for the investigated barcoding region are shown in Table 2. *Mat*K1F/2R oligos were used in *Cedrus* (Wang et al. 1999). PCR products were cleaned with Illustra DNA/Gel Band Purification Kit (GE Healthcare). Standard aliquots were submitted to Macrogen Inc. (http://www.macrogen.com) for sequencing. Electropherograms were edited with CHROMAS 2.3 (http://www.technelysium.com.au) and checked visually.

**Table 2.** Primers list.

Marker region	Primers	Reference
***rbc*L**	Fw - ATGTCACCACAAACAGAAAC	Kress et al. (2005)
	Rev - TCGCATGTACCTGCAGTAGC	
**trnH-psbA**	Fw - CGCGCATGGTGGATTCACAATCC	Shaw et al. (2007)
	Rev - GTTATGCATGAACGTAATGCTC	
***mat*K_Kim**	Fw - CGTACAGTACTTTTGTGTTTACGAG	Kim (unpublished)
	Rev - ACCCAGTCCATCTAAATCTTGGTTC	
***mat*K1F/2R**	Fw - GAACTCGTCGGATGGAGTG	Wang et al. (1999)
	Rev - TAAACGATCCTCTCATTCACGA	

### Bioinformatics tools

Sequences were aligned with MEGA5 (Tamura et al. 2011) and checked by eye. Haplotypes were defined with BLASTClust v2.2.20 (http://toolkit.tuebingen.mpg.de/blastclust) with the following command line: blastclust -i infile -o outfile -p F -L1 -bT -S100, thus requiring to cluster together only sequences with 100% identity and length coverage. All the species presenting single haplotypes were considered efficiently discriminated; those displaying at least one haplotype in common with another species were considered precluded to discrimination.

Species discrimination power of the investigated loci was also assessed using the genetic distance approach, to evaluate whether the amount of variation displayed was sufficient to discriminate sister species without affecting their correct assignation through intraspecific variation. This approach is at the basis of the “barcoding gap” definition, i.e. the assumption that the amount of sequence divergence within species is smaller than that between species. Uncorrected p-distance matrices of sequence divergences within and among congeneric species were calculated for each gene fragment and for the two joined markers (*rbc*L + trnH-psbA), with MEGA5. All the species presenting a minimum interspecific distance value higher than their maximum intraspecific distance were considered successfully discriminated (Meyer et al. 2008).

Finally, we simulated a barcode identification scenario using each sequence as an unknown query and GenBank (http://www.ncbi.nlm.nih.gov) as global reference database. The NCBI Taxonomy database (http://www.ncbi.nlm.nih.gov/taxonomy) was screened to assess the presence of the investigated species set in GenBank, relatively to markers under study. The identification ability of every single marker was evaluated using the megaBLAST algorithm (http://blast.ncbi.nlm.nih.gov) with default parameters and adjusted to retrieve 5000 sequences. A query sequence was considered as successfully identified if the top Bit-score obtained in GenBank matched the name of the species (Ross et al. 2008). Identification success was only inferred for species/sequences represented in GenBank. When more than one species shared a top Bit-Score or the species scored lower, the result was considered an identification failure.

## Results

### Markers’ main features

Optimal amplification rates were obtained with *rbc*L and trnH-psbA which produced clear, single-banded PCR products from all 68 investigated samples (136 sequences; 100% efficiency). *Mat*K was not consistently amplified in the Pinaceae and Rosaceae (44.1% of the investigated dataset) and thus it was not included in further analyses. All *rbc*L electropherograms were easily read and analysed. Conversely, the very long poly-nucleotide repeatsin the trnH-psbA regions of *Sambucus* sp. made subsequent traces hardly readable. Consequently, in this genus the entire sequences were completed by joining partial bidirectional reads (Kress and Erickson 2007). The alignment of *rbc*L sequences was straightforward with a consensus of 688 bp (no indels found). The trnH-psbA sequences varied greatly in length, ranging from 396 (*Sorbus* and *Crataegus* spp.) to 622 bp (*Cedrus* spp.). Numerous gaps were observed in this region. An indel of 45 bp turned out to be diagnostic to discriminate the two *Aesculus* species, an indel of 55 bp discriminated *Fraxinus ornus* from *Fraxinus excelsior* and *Fraxinus angustifolia*, one of 66 bp discriminated *Sambucus ebulus* from *Sambucus racemosa* and other indels (20-22 bp) were diagnostic for *Sorbus torminalis* and *Cedrus deodara*. Shorter gaps (1-19 bp) were detected intraspecifically in all species except in *Punica*, *Ziziphus* and *Ilex*. All sequences have been deposited in GenBank under accession numbers HG765031-HG765098 (*rbc*L), and HG764963-HG765030 (trnH-psbA).

### Markers’ discrimination ability

The alignment–free method implemented in BLUSTClust produced for each marker the haplotypes shown in Table 3. Based on the uniqueness of sequence character states, trnH-psbA generated a total of 43 haplotypes, 35 of which could be ascribed to single species. Common haplotypes were displayed by 14 individuals of the following species pairs, thus preventing their discrimination: *Fraxinus angustifolia*–*Fraxinus excelsior* (three samples), *Crataegus monogyna*–*Crataegus oxyacantha* (four samples), *Sorbus aucuparia*–*Sorbus domestica* (two samples), *Ilex aquifolium*–*Ilex latifolia* (five samples). Consequently, trnH-psbA discrimination ability was 79.4% of the investigated plants, corresponding to 66.7% of the species in the total dataset, 63.6% considering only those genera in which at least one species pair was sampled.

**Table 3.** Haplotypes generated by BLASTClust in the investigated dataset with both markers and their combination. Shaded: species where unique haplotypes (either single or in combination) were detected.

Species	Samples	Unique haplotypes			Inter-species shared haplotypes		
		*rbc*L	trnH-psbA	Combined	*rbc*L	trnH-psbA	Combined
*Cedrus atlantica*	3	2	2	2	/	/	/
*Cedrus deodara*	3	1	1	1	/	/	/
*Cedrus libani*	3	1	1	1	/	/	/
*Crataegus monogyna*	3	/	/	/	1	1	1
*Crataegus oxyacantha*	2	/	1	1	1	1	1
*Crataegus azarolus*	4	/	2	2	1	/	/
*Sorbus aria*	3	1	3	3	/	/	/
*Sorbus aucuparia*	2	1	1	1	1	1	1
*Sorbus domestica*	3	/	1	1	1	1	1
*Sorbus torminalis*	3	1	1	1	/	/	/
*Aesculus hippocastanus*	3	1	2	2	/	/	/
*Aesculus indica*	3	1	3	3	/	/	/
*Fraxinus ornus*	5	2	4	5	1	/	/
*Fraxinus angustifolia*	3	/	1	1	1	1	1
*Fraxinus excelsior*	2	/	/	/	1	1	1
*Sambucus nigra*	5	1	4	4	1	/	/
*Sambucus ebulus*	2	1	2	2	1	/	/
*Sambucus racemosa*	1	1	1	1	/	/	/
*Passiflora incarnata*	2	2	2	2	/	/	/
*Passiflora edulis*	1	1	1	1	/	/	/
*Punica granatum*	4	1	1	1	n.d.	n.d.	n.d.
*Ziziphus jujuba*	3	1	1	1	n.d.	n.d.	n.d.
*Ilex aquifolium*	4	/	/	/	1	1	1
*Ilex latifolia*	1	/	/	/	1	1	1
**Total**	**68**	**19**	**35**	**36**	**12**	**8**	**8**

*Rbc*L displayed a much lower sequence differentiation (with a total of 31 haplotypes, 12 of which were shared between species). No haplotypes were shared among species from different genera. The two-marker combination did not improve markedly the discrimination efficacy displayed by trnH-psbA alone.

In this study, the two potential DNA barcodes displayed different levels of intra- and inter-speciﬁc distances. With *rbc*L, all intra-speciﬁc uncorrected p-distances were zero, except in *Cedrus atlantica* (0.0014), *Sorbus aria* (0.0014), *Sorbus aucuparia* (0.0028), *Crataegus monogyna* (0.0028), and *Sambucus ebulus* (0.004). Zero inter-speciﬁc distances were detected between individuals belonging to *Sorbus aucuparia* and *Sorbus domestica*, among the three *Crataegus* species, the three *Fraxinus* species, between *Sambucus nigra* and *Sambucus ebulus*, and between the two *Ilex* species. Conversely, no intraspecific sequence variation was found at trnH-psbA in *Cedrus deodara*, *Cedrus libani*, *Sorbus torminalis*, *Crataegus monogyna*, *Crataegus oxyacantha*, *Fraxinus angustifolia*, *Sambucus racemosa*, *Passiflora edulis*, *Punica granatum*, *Ziziphus jujuba* and the two *Ilex* species. Inter-speciﬁc genetic differences produced by this marker exhibited values higher than zero (0.0018–0.0298) only in five species belonging to *Cedrus*, *Aesculus* and *Passiflora* genera, and in *Fraxinus ornus* and *Sambucus racemosa*.

The values of the maximum intra- and minimum interspecific sequence divergence of the two combined barcoding loci are shown in Table 4  (all inter-speciﬁc distances involve congeneric species). In agreement with data based on the single markers, non-overlapping intra- and interspecific distances were observed in a few species groups. As such, barcoding gaps were observed in *Cedrus deodara* and *Cedrus libani*, *Sorbus torminalis*, and the two *Aesculus* species. All remaining taxa displayed equal (e.g. in *Cedrus atlantica*) or higher values of intra- than interspecific divergence (e.g. in *Passiflora incarnata*, *Fraxinus ornus*, *Sorbus aria*). Several species showed sequences involving zero interspecific divergence (e.g. *Sorbus domestica*, *Sorbus aucuparia*, *Fraxinus excelsior*, *Fraxinus angustifolia*, *Sambucus nigra*, *Sambucus ebulus*, *Crataegus* spp.). The lack of additional conspecific samples did not allow a comparison with the high levels of inter-speciﬁc divergences shown by two species (*Passiflora edulis* and *Sambucus racemosa*). These results suggest that there is a barcoding gap in only five out of 19 analyzed species, corresponding to 26.3% of our dataset (taxa with only one individual/species or one species/genus excluded).

**Table 4.** Values of maximum inter- and minimum intraspecific uncorrected p-genetic distances resulting from the combination of *rbc*L + trnH-psbA sequences, and relative barcoding gaps calculated in 24 forest tree taxa; n.d. = not determined; * = no sister species included in the dataset; ** = *taxa* with single accession. Shaded: species where a barcoding gap was detected.

	Samples	Max. Intrasp. distance	Min Intersp. distance	Barcoding gap
*Cedrus atlantica*	3	0.0015	0.0015	0
*Cedrus deodara*	3	0	0.0015	0.0015
*Cedrus libani*	3	0	0.0023	0.0023
*Sorbus aria*	3	0.002898554	0.000950571	-0.0019
*Sorbus aucuparia*	2	0.0058	0	-0.0058
*Sorbus domestica*	3	0.0009	0	-0.0009
*Sorbus torminalis*	3	0	0.0009	0.0009
*Crataegus azarolus*	3	0.0009	0	-0.0009
*Crataegus monogyna*	2	0.0019	0	-0.0019
*Crataegus oxyacantha*	4	0	0	0
*Aesculus hippocastanus*	3	0	0.0064	0.0064
*Aesculus indica*	3	0	0.0064	0.0064
*Fraxinus ornus*	5	0.00568	0.00284	-0.0028
*Fraxinus angustifolia*	3	0.0036	0	-0.0036
*Fraxinus excelsior*	2	0	0	0
*Sambucus nigra*	5	0.0017	0	-0.0017
*Sambucus ebulus*	2	0.0101	0	-0.0101
Sambucus racemosa**	1	n.d.	0.0142	n.d.
*Passiflora incarnata*	2	0.02397	0.01588	-0.0081
Passiflora edulis**	1	n.d.	0.0158	n.d.
Punica granatum*	4	0	n.d.	n.d.
Ziziphus jujuba*	3	0	n.d.	n.d.
*Ilex aquifolium*	4	0	0	0
Ilex latifolia**	1	n.d.	0	n.d.

The NCBI Taxonomy database screening revealed that all the species in our dataset were represented by *rbc*L and trnH-psbA marker sequences in the database, except for *Aesculus indica*, *Cedrus libani* (neither marker), *Crataegus azarolus* and *Sorbus domestica* (only *rbc*L present).

When BLASTed to GenBank, all our *rbc*L sequences were identified by the reference sequences at the genus level (87.5% of total taxa), or even at the species level (41.6%). Genus misidentification occurred in the three *Crataegus* species, for which genera *Cotoneaster*, *Pyrus*, *Piracantha*, *Amelanchier*, *Chaenomeles* (all belonging to the Rosaceae family) and *Crataegus* were also the best match. In contrast, correct genus and species identifications were obtained for *Ilex aquifolium*, *Passiflora incarnata* and *Passiflora edulis*, *Punica granatum*, *Ziziphus jujuba*, *Sambucus nigra*, *Sorbus torminalis*, *Cedrus atlantica* and *Cedrus deodara*.

TrnH-psbA was outperformed by *rbc*L, since none of the *Sorbus* sequences (four species) matched the right genus, and only eight species (33.3%) were correctly identified (*Fraxinus ornus*, *Passiflora incarnata*, *Punica granatum*, *Ziziphus jujuba*, *Sambucus racemosa*, *Cedrus atlantica* and *Cedrus deodara*). All other samples shared the highest score with other species (e.g. *Aesculus hippocastanum* with *Aesculus turbinata*, *Fraxinus excelsior* with *Fraxinus angusitfolia*, *Sambucus nigra* with *Sambucus racemosa*, *Crataegus monogyna* with several other species), or even hit the wrong species (e.g. *Ilex aquifolium*, *Sambucus ebulus*, *Crataegus oxyacantha*). The four taxa not represented in GenBank (*Cedrus libani*, *Aesculus indica*, *Creataegus azarolus* and *Sorbus domestica*) were assigned to the correct genus. As a final result, only 11 species were correctly identified by the two locus-combination corresponding to 55% of the investigated species having a reference in GenBank (45.8% of the total species set). A summary of the correct species identifications achieved with the three discrimination methods used in the present study is shown in Table 5. Thirteen species (54.2% of our dataset) were identified by at least two methods. Only two species (*Cedrus deodara* and *Sorbus torminalis*) were identified with the three methods, whereas the absence of conspecific GenBank references prevented the same full identification for *Cedrus libani* and *Aesculus indica*. In contrast, six species (corresponding to three species pairs and totalling 25% of our dataset) appeared unidentifiable with any method: *Crataegus monogyna*, *Crataegus oxyacantha*, *Sorbus aucuparia*, *Sorbus domestica*, *Fraxinus angustifolia*, *Fraxinus excelsior*. Two species (*Crataegus azarolus* and *Sorbus aria*) were discriminated only by means of sequence specificity but received no confidence by any of the other two approaches (the former was absent in GenBank).

**Table 5.** Summary of the species identification success achieved with *rbc*L + trnH-psbA and the three discrimination methods in the present study: occurrence of unique haplotypes in the total species set, genetic distances among and within congeneric species, correct species match in the GenBank database. Green: correct identification; red: non confident/wrong identification; shaded = not determined (no intra- or interspecific samples investigated); a = species absent in GenBank with either one or both markers.

Species	Identification success		
	Haplotype specificity	Min. inter- > max. intraspecific distance	GenBank correct match
*Cedrus atlantica*	**√**	-	**√**
*Cedrus deodara*	**√**	**√**	**√**
*Cedrus libani*	**√**	**√**	**a**
*Crataegus monogyna*	-	-	-
*Crataegus oxyacantha*	-	-	-
*Crataegus azarolus*	**√**	-	**a**
*Sorbus aria*	**√**	-	-
*Sorbus aucuparia*	-	-	-
*Sorbus domestica*	-	-	**a**
*Sorbus torminalis*	**√**	**√**	**√**
*Aesculus hippocastanus*	**√**	**√**	-
*Aesculus indica*	**√**	**√**	**a**
*Fraxinus ornus*	**√**	-	**√**
*Fraxinus angustifolia*	-	-	-
*Fraxinus excelsior*	-	-	-
*Sambucus nigra*	**√**	-	**√**
*Sambucus ebulus*	**√**	-	-
*Sambucus racemosa*	**√**	n.d.	**√**
*Passiflora incarnata*	**√**	-	**√**
*Passiflora edulis*	**√**	n.d.	**√**
*Punica granatum*	**√**	n.d.	**√**
*Ziziphus jujuba*	**√**	n.d.	**√**
*Ilex aquifolium*	-	-	**√**
*Ilex latifolia*	-	n.d.	-
**Efficacy**	**66.7%**	**26.3%**	**55%**

## Discussion

### Marker applicability

In our dataset, the *rbc*L + trnH-psbA combination showed the highest amplification and sequencing success (100%), whereas *mat*K showed a much lower success (55.9%). Specifically, the currently most adopted primers set for Angiosperms (*mat*K_KIM) failed in the amplification of the Rosaceae, and *mat*K1F/2R primers, suggested for the Pinaceae, failed to amplify *Cedrus* sp. In addition, *mat*K also revealed severe difficulties in the amplification and/or sequencing steps in the genera *Berberis* (Berberidaceae), *Vitex* (Rhamnaceae), *Cercis* (Leguminosae) and *Ginkgo* (Ginkgoaceae), in the ongoing prosecution of this work. The lack of universality of *mat*K was already reported by e.g. Kress and Erickson (2007), Fazekas et al. (2008), Ford et al. (2009), De Mattia et al. (2012). MatK_KIM, (Kim, unpublished) is still considered the primer set with the highest match for eudicots, while *mat*K1F/2R was efficiently used in a comprehensive study across Pinaceae (Wang et al. 1999). Dunning and Savolainen (2010) also noted that *mat*K_KIM is not the best choice for Rosaceae and rather suggested the use of specific primer sets. The difficulty of defining the best primer choice for *mat*K in Conifers was already faced by e.g. Li et al. (2011) and Armenise et al. (2012). When applied to international trade and safe use of medicinal plants, *mat*K yielded 54.0% of amplification efficiency in Chen et al. (2010), whereas Kool et al. (2012) produced PCR products for less than 30% of the specimens, and sequencing success was only 10% in Wallace et al. (2012).

In contrast, trnH–psbA provided better discrimination than *mat*K in many diverse tree genera such as *Alnus* (Roy et al. 2010), *Ficus* (Ren et al. 2010), *Quercus* (Simeone et al. 2013), and more generally in Angiosperms (Pang et al. 2012). Nevertheless, *mat*K is still recommended by the CBOL Plant working Group (2009) as the first option to rely on in terms of sequence variability. We therefore suggest that an efficient barcoding workflow should include a first preliminary screening with *mat*K universal primer set(s) and then, depending to the amplification results, to select trnH-psbA as an additional marker to *rbc*L. Alternatively, a simple and clear morphological trait may be included in the analysis or address the search for the most appropriate *mat*K primer set based on the biological group under study (Bruni et al. 2012, Dunning and Savolainen 2010).

### Species identification and discrimination

The BLUSTClust analysis yielded a 66.7% species discrimination, which is a bit lower but still in line with the general limit acknowledged for land plants when markers from a single genetic linkage group are used (ca. 70%; CBOL Plant Working Group 2009). In agreement, similar percentages (68–71%) were obtained in broader taxonomic investigations in forests of North and meso-America (Fazekas et al. 2008, Gonzalez et al. 2009), although by use of a different way to assess species identification success (i.e. support for species monophyly through barcodes). Our barcoding data, dedicated to woody plants sampled in a different ecological zone, approach Piredda et al. (2011), who reported 73% efficiency in a floristic investigation of the Italian tree flora by means of sequence specificity; nevertheless, more intraspecific diversity and more species pairs were surveyed in the present work.

The highest identification success was achieved with the analysis based on the uniqueness of sequence character states, where some parts in the haplotypes (especially some trnH-psbA indels) appeared diagnostics for certain species. However, more data are required to confirm these diagnostic sequence features. Yet, if confirmed, these features may be important in view of the generally low interspecific divergences we observed. Conversely, the analysis with the barcoding gaps suggests that such a discrimination approach may yield a lower efficiency, at least with trnH-psbA, since the uncorrected p-distance analysis removed all indels. A further complication we encountered was constituted by the high intraspecific divergences (e.g. in *Cedrus atlantica*) and the sharing of haplotypes among congeneric species (e.g. in *Sorbus*, *Crataegus*, *Fraxinus*, *Sambucus*). All these results challenge the application of DNA barcoding with *rbc*L + trnH-psbA in the taxa investigated here. This is the more so as GenBank also showed a low identification efficiency and sometimes lead to erroneous identifications, most often due to the limited number of available reference sequences and their sometimes very high intraspecific divergences. Little and Stevenson (2007) and Ross et al. (2008) found that BLAST (and other similarity methods) can give accurate identifications on GenBank (see also de Vere et al. 2012 and Pang et al. 2012), although some distorted results, in inverse proportion to the number of reference sequences per species in the databases, may render these approaches inappropriate. Ideally, a reference library should provide multiple samples from unambiguously identified species or taxa, and cover intraspecific variability and closely related species to evaluate the degree of divergences among barcodes. Unfortunately, the reference list in the GenBank database is still far from complete. The small numbers of available sequences per species and for either marker prevented us from confidently retrieving correct species names in *Aesculus hippocastanum*, *Fraxinus excelsior*, *Ilex latifolium*, *Crataegus monogyna* (highest scores shared with other congenerics). Moreover, it induced us to assign a query to the wrong species, as in the cases of *Aesculus indica* (*Aesculus pavia*), *Fraxinus angustifolia* (*Fraxinus excelsior*), *Passiflora edulis* (*Passiflora incarnata*), *Sambucus ebulus* (*Sambucus adnata*), *Crataegus azarolus* and *Crataegus oxyacantha* (*Crataegus monogyna*), *Cedrus libani* (*Cedrus deodara*), and the four *Sorbus* species. Clearly, a consistent enrichment of the reference databases is a priority for future applications of DNA barcoding.

### DNA barcoding of medicinal and aromatic plants

DNA barcoding is a substantial improvement of our capacity to document the existing biodiversity. It is also a powerful research complement for human socio-economics, safety, trade control, frauds discovery and detection of forgeries in plant commercial products (Newmaster and Ragupathy 2010). Kool et al. (2012), for example, were able to document 18 misidentifications and eight forgeries among 111 samples of medicinal plants in a local market in Marrakech (Morocco).

The Mediterranean woody flora comprises numerous valuable species used as ornamentals or for secondary products processing and marketing (edibles, essential oils, medicinal compounds). Field identification, authentication and certification of germplasm and raw materials are a major concern. As such, our results on *Cedrus* support previous findings that members of Pinaceae can be efficiently barcoded with *rbc*L + trnH-psbA (at least at a regional scale; Armenise et al. 2012). Cedars involve four different extant species: the three more highly diffused and with great ornamental, ecological and cultural relevance were here discriminated, while *Cedrus brevifolia*, a highly protected, rare endemic surviving in only one population on Troodos Mountains (Cyprus), still awaits further investigations. We also found specific haplotypes for the highly important and largely cultivated *Punica granatum*. In this case as well, further investigations involving the only other species of genus *Punica* (*Punica protopunica*, a rare endemic of the Socotra Island, Yemen, very similar in morphology, production of fruits and secondary metabolites) would eventually provide new tools for its conservation and management.

On the other hand, we confirm the difficulties previously encountered in barcoding *Fraxinus* (Arca et al. 2012) and the extensive interspecific haplotype sharing in *Crataegus* (Fineschi et al. 2005) and *Sorbus* (Robertson et al. 2010). For instance, Burgess et al. (2011) were able to discriminate only one out of four *Crataegus* species with five barcoding markers. Indeed, these genera are likely to be as refractory to barcoding as other woody groups including oaks (Piredda et al. 2011) and willows (von Crautlein et al. 2011). Low mutation rates, incomplete lineage sorting and hybridization are the most reported causes (Hollingsworth et al. 2011). However, we were able to discriminate *Fraxinus ornus*, a very important medicinal and industrial plant, and *Crataegus azarolus*, a protected fruit tree, historically used for a number of medicinal purposes. Conversely, we were unable to discriminate the *Crataegus monogyna* – *Crataegus oxyacantha* species pair (see also Bruni et al. 2012), but this has little practical importance since both hawthorns are equally used for the same medicinal purposes. Very promising data were collected on *Sorbus aria* and *Sorbus torminalis*, *Ilex aquifolium*, *Aesculus Hippocastanum*, *Passiflora* and *Ziziphus jujuba*, suggesting that an efficient barcoding could be achieved on these species, at least at regional scales. In contrast, *Sambucus* sp. showed a large intraspecific divergence and require further investigations on larger datasets. More recently, the nuclear ribosomal ITS (especially the ITS2 portion) has been suggested as an efficient barcoding locus for complex plant groups (Chen et al. 2010). However, Kool et al. (2012) could not use this marker in 45% of their dataset because of the low amplification and sequencing efficacy detected and fungal contamination, particularly in the root material. Therefore, this marker still appears not completely devoid of some pitfalls and certainly will require an improvement of current protocols.

## Conclusion

Recently, an outstanding research interest towards DNA barcoding of regional floras with biological and/or economical relevance has spread. In the present work, we lay the foundations towards DNA barcoding applications of important woody plant genera in the Mediterranean basin, such as *Cedrus*, *Aesculus*, *Ilex*, *Passifllora*, *Punica*, *Sambucus*, *Sorbus*, *Ziziphus*. All these genera include valuable taxa for multiple natural and economic purposes, and combine with similar DNA barcoding investigations performed on Euro-Mediterranean forested land in recent years (Piredda et al. 2011, von Crautlein et al. 2011, Armenise et al. 2012, Simeone et al. 2013). Gathered results expose limitations of DNA barcoding, most of which are due to (1) the imperfect discrimination ability of the markers and methods currently in use, (2) the biological peculiarities of some genera, and (3) the low taxonomic coverage of the reference databases. Future technological advances, additional markers and larger sample sets at different geographical scales (from continental to local) are therefore auspicated to improve current protocols and identification success for the practical conservation and valorisation of forest natural resources.
